# Treatment of Severe Vesical Inflammation in the Female

**Published:** 1909-08

**Authors:** 


TREATMENT OF SEVERE VESICAL INFLAMMATION IN THE FEMALE.

E. ZURHELLE (Fritsch's University Gynecological Clinic at Bonn) ; *Zeitschr. f. gynäk. Urologie,* 1908, No. 2, says that: silver nitrate, even in weak solution and skilfully used, is often painful. When the bladder is irritated more intensely by a silver irrigation the burning becomes so violent that soothing remedies must be resorted to. Only in some of the cases can the remaining solution be left in the bladder for any length of time; generally the contents must be voided after a few minutes. Incited by the publication of Voelcker and Lichtenberg (Czerny's clinic) I have therefore for the past one and a half years used collargol in place of silver nitrate. In a series of chronic cystitides, some of them desperate, I injected 3½ ounces of lukewarm 1 per cent. solution, after washing out and emptying the bladder. The absolute absence of any irritative symptom made it possible to leave the solution in the bladder many hours, the effect being correspondingly more vigorous. In fact, the effect seems to be a lasting one. In several cases, after cessation of treatment for some days, I found cystoscopically that there was an abundant precipitate of collargol over the entire mucosæ of the vesical wall. I do not think we can obtain a similar lasting effect with any other means. And this without any irritation!

In very severe cases, hitherto treated with silver nitrate without avail, the injection of collargol proved effectual in ten to twelve days, in milder cases within a week. It is certainly preferable to the instillation of a few drops of concentrated (1 per cent. silver nitrate solution, from which others too have seen but little success and much pain. How the therapeutic effect of collargol in the bladder is to be explained, whether due to the catalytic properties which it owes to its colloidal character and which may by oxidation processes transform poisonous bacterial products into inert bodies, I do not feel able to say. But the fact of good effect is established.

The decrease in our post-operative cystitides is probably due to the fact that we catheterise as little as possible. Perhaps the prophylactic filling of the bladder with collargol after operation has rendered cystitis more rare.

I think the method outlined not only will very often shorten the course of severe inflammations of the femals bladder, but in isolated cases may avert the last resort, namely, the need of making a vesical fistula. Collargol can do good service especially in the cases in which the contractile power of the bladder is so much reduced that after spontaneous urination there always remains a residual urine with stagnation and decomposition, which maintains the cystitis and which hitherto could be coped with only by the use of constant catheterisation. Of course collargol is no panacea for cystitis.

Addendum: I am glad to see in the report of *Stoeckel* in the last number of this publication that he has "found collargol solution in the treatment of cystitis extremely satisfactory."

Drs. Moncorvo and Pires (*Med. de los Ninos,* July, 1908,) on the basis of an experience of thirty-four cases, recommend in all cases of infantile dysentery the use of collargol enemata preceded by a cleansing irrigation. All their cases recovered under the treatment.

